# Cardiac Rehabilitees’ Technology Experiences Before Remote Rehabilitation: Qualitative Study Using a Grounded Theory Approach

**DOI:** 10.2196/10985

**Published:** 2019-02-07

**Authors:** Marjo-Riitta Anttila, Heikki Kivistö, Arja Piirainen, Katja Kokko, Anita Malinen, Mika Pekkonen, Tuulikki Sjögren

**Affiliations:** 1 Faculty of Sport and Health Sciences University of Jyväskylä Jyväskylä Finland; 2 Faculty of Education and Psychology University of Jyväskylä Jyväskylä Finland; 3 Peurunka Rehabilitation Laukaa Finland

**Keywords:** coronary disease, rehabilitees’ experience, focus group, qualitative study, grounded theory, remote rehabilitation, e-health, e-rehabilitation, telerehabilitation, secondary prevention

## Abstract

**Background:**

Even though technology is becoming increasingly common in rehabilitation programs, insufficient data are as yet available on rehabilitees’ perceptions and experiences. It is important to understand their abilities when using technology for remote rehabilitation.

**Objective:**

This is a qualitative study on technology experiences of persons affected by cardiovascular disease assessed before remote rehabilitation. The aim of the study was to explore rehabilitees’ experiences and attitudes toward technology before 12 months of remote rehabilitation.

**Methods:**

Qualitative interviews were conducted with 39 rehabilitees in four focus groups. The subjects were aged 34 to 77 years (average age 54.8 years) and 74% (29/39) of them were male. They had been diagnosed with coronary artery disease and were undergoing treatment in a rehabilitation center. The interviews were conducted between September 2015 and November 2016. Data were analyzed using Glaser’s mode of the grounded theory approach.

**Results:**

The result of the study was an “identifying e-usage” experience category, which refers to the rehabilitees’ notions of the use of information and communication technologies (e-usage) in the process of behavior change. The main category comprises four subcategories that define the rehabilitees’ technology experience. These subcategories are “feeling outsider,” “being uninterested,” “reflecting benefit,” and “enthusiastic using.” All rehabilitees expected that technology should be simple, flexible, and easy to use and learn. The results reflecting their technology experience can be used in e-rehabilitation programs. Rehabilitees who feel like outsiders and are not interested in technology need face-to-face communication for the major part of rehabilitation, while rehabilitees who reflect benefit and are enthusiastic about the use of technology need incrementally less face-to-face interaction and feel that Web-based coaching could offer sufficient support for rehabilitation.

**Conclusions:**

The findings show that persons affected by heart disease had different experiences with technology and expectations toward counseling, while all rehabilitees expected technology to be easy to use and their experiences to be smooth and problem-free. The results can be used more widely in different contexts of social and health care for the planning of and training in remote rehabilitation counseling and education.

**Trial Registration:**

ISRCTN Registry ISRCTN61225589; http://www.isrctn.com/ISRCTN61225589 (Archived by WebCite at http://www.webcitation.org/74jmrTXFD)

## Introduction

Cardiovascular diseases are the most common cause of death globally [1]. Cardiac rehabilitation is a means of secondary prevention intervention for cardiovascular diseases that includes efforts to reduce behavioral risks such as tobacco use, unhealthy diet, obesity, physical inactivity, alcohol use, and psychosocial problems such as depression [1,2]. However, many persons with coronary artery disease are not aware of opportunities to participate in rehabilitation programs or they choose not to participate in cardiac rehabilitation for a number of reasons, including living a long distance from a facility [3,4]. It is necessary to develop new methods of rehabilitation such as Web-based programs [5]. With global digitalization, rehabilitation increasingly uses technology. Remote rehabilitation programs use a range of remote technologies and Web-based applications. Remote counseling means professionally coached and monitored rehabilitation with a clearly defined beginning and end [6-8]. Digital eHealth tools include wireless digital devices like mobile phones and tablet computers, self-care and self-monitoring devices, video call services such as Skype for Business, wearable and ingestible sensors and various digital applications, and virtual reality made possible by robots and other forms of new technology [9]. Digitalization requires new attitudes and skills from rehabilitees and professionals [10].

The use of remote technology in cardiac rehabilitation has been studied mainly by quantitative methods [11]. Research has focused on the effectiveness [6] and usability [12] of technology-intensive interventions. Issues related to rehabilitees’ physical activity [6,13,14] and lifestyle change [8,15] have been another focus area. Qualitative studies are a minority, and they have focused on experiences of participation in Facebook peer groups [16,17], mHealth [18], eHealth [19], or Web-based programs [20-22].

However, research has rarely looked at the role of remote technology in cardiac rehabilitation [14,23]. Research is needed to expand the understanding of the experiences of persons who use or have used remote technology and assess the pros and cons of this technology [7,8,11]. The aim of this study was to gain an understanding of cardiovascular rehabilitees’ experiences with technology and of their attitudes toward technology.

## Methods

### Study Approach

We used a grounded theory approach in this study. Data were analyzed using Glaser’s inductive grounded theory approach. We decided to apply a methodology proposed by the grounded theory approach because we found grounded theory useful in getting to understand the rehabilitees’ subjective experiences for generating a substantive theory in a relatively new research area [24]. The focus was on finding out the rehabilitees’ experiences and attitudes toward technology prior to using remote technology. The rehabilitees described in qualitative interviews their experiences with computer use, social media, and other applications of modern technology.

### Recruitment

The interviews were conducted in 2015 and 2016 in Rehabilitation Centre Peurunka, Finland, where the Social Insurance Institution of Finland arranges regular cardiac rehabilitation courses. The study is a part of a remote technology in cardiac rehabilitation study registered at the ISRCTN Registry [ISRCTN61225589]. The Ethics Committee of the Central Finland Health Care District approved the study.

### Participants

The participants were 39 rehabilitees (10 women and 29 men); 82% (32/39) of them had undergone coronary angioplasty and 10% (4/39) had undergone coronary artery bypass about 3 to 12 months prior to rehabilitation. Most subjects had a computer (23/27, 85%) and used the internet (25/27, 92%). Many had mobile phones (16/27, 59%) and tablets (10/27, 37%), and several used wrist activity trackers (10/27, 37%). These statistics are similar to those obtained during the testing of other European cardiac patient populations [25]. According to Glaser’s inductive grounded theory approach, baseline information and characteristics of the subjects were collated later for this paper and were not taken into account in the analysis [24] (Table 1).

### Data Collection

The total duration of rehabilitation was 15 days spread between three 5-day periods over a time span of 12 months. Rehabilitation took place in a rehabilitation center [26]. Qualitative interviews were conducted at the beginning of rehabilitation in 4 focus group discussions, each interview lasting 30 to 60 minutes, overall 156 minutes. The interviews were conducted by the same interviewer and they were informal and semistructured. The questions were mostly unplanned and spontaneous, but the interviewer also presented the same series of open-ended questions to all subjects. They were asked questions like, “Tell me about your experience with modern technology,” and “What are your expectations of remote counseling?” The interviews were audio-recorded and transcribed for analysis. The transcripts were imported into ATLAS.ti (ATLAS.ti Scientific Software Development GmbH) computer software, which enables data storage, organization, and retrieval for analysis. The number of subjects was determined according to data saturation, which is a point at which no more new experiences of the topic could be elicited [24].

**Table 1:** Description of participants.

Characteristic		Under 55 years, n (%)	55 years and over, n (%)	Total, n (%)
Age (years)		20 (51)	19 (49)	39 (100)
**Gender**				
	Male	17 (85)	12 (63)	29 (74)
**Education**				
	Vocational or course-form school or other	13 (68)	14 (74)	27 (71)
	College-level education	3 (16)	3 (16)	6 (16)
	University of applied sciences	2 (11)	2 (11)	4 (11)
	University	1 (5)	0 (0)	1 (3)
**Time of operation**				
	0-3 months from rehabilitation	1 (5)	0 (0)	1 (3)
	3-12 months from rehabilitation	13 (65)	11 (58)	24 (62)
	Over 12 months from rehabilitation	4 (20)	6 (32)	10 (26)
	No operations	2 (10)	2 (11)	4 (10)
**Technology**				
	Internet, yes	12 (100)	13 (87)	25 (93)
	Computer, yes	11 (92)	12 (80)	23 (85)
	Tablet computer, yes	6 (50)	4 (27)	10 (37)
	Mobile phone, yes	7 (58)	9 (60)	16 (59)
	Physical activity tracker, yes	4 (33)	6 (40)	10 (37)

### Data Analysis

The constant comparative model guided the data collection process. Data were collected through 4 informal interviews and analyzed using the constant comparative model. We collected and analyzed data concurrently, and as the analysis progressed the research question became more focused. First, we started substantive coding [24,27]. Subcategories were created in open coding. We analyzed incidents and compared them with other incidents, looking for similarities and differences and creating as many concepts as possible, coding substantively. Being theoretically sensitive, data were closely read and questioned. Next, we identified the properties and dimensions of each subcategory. Finally, we grouped the concept into subcategories creating as many subcategories as possible and then integrated the subcategories into the category. During this constant comparative model process, we recorded our ideas and notions, which helped us process the data. Data analysis was continued until the category was theoretically saturated [24].

The following example describes the creation of the “being uninterested” subcategory. The analysis began with open coding. We analyzed data on the diversity of the rehabilitees’ experiences of technology use as well as their attitudes toward, and expectations for, remote counseling. This perspective expanded from their responses and debates. Constant comparison convinced us that all codes essentially described or explained how each rehabilitee used technology or what their attitude toward technology was. Next we named properties (1) using technology occasionally, (2) limiting to use, (3) challenging problem-free technology, and (4) activating empowerment counseling. We named the subcategory for this experience as “being uninterested.” Textbox 1 shows an example of the process.

The constant comparison of properties resulted in hypotheses about relationships between the subcategories [28]. We continued to collect and analyze data until no new subcategories emerged and the subcategories were saturated; a category was thereby created and named “rehabilitees identifying e-usage in the process of behavior change.”

**Creating category for e-usage identification.**

Category:

- Identifying e-usage

Subcategory:

- Being uninterested

Property:

- Using technology occasionally
- Limiting to use
- Challenging problem-free technology
- Activating empowerment counseling

Concepts (code):

- The rehabilitee is not interested in using technology in his free time.
- Being bound to technology irritates; the rehabilitee does not want to use technology all the time.
- Ineffective technology worries the rehabilitee.
- The rehabilitee waits to be communicated with by email.
- The rehabilitee expects counseling to be a motivator and to spark interest

Text:

- I’m not terribly interested in that remote stuff, because...well, I use the computer at work every day....That email reading, I may go and check my mail once a week. It’s not in a way...maybe it just isn’t my thing...it’s no trouble to surf on the internet in the evening. Only when I must. I can check email—if a bill has arrived, I can pay it there. [Participant 9, 44-year-old man, focus group 2]
- ...but I don’t like that one’s got to be, like, twenty-four hours a day available. [Woman, 59 years, focus group 4I’ve noticed sometimes that when I’ve been at it for some time, the machine has broken down in the middle of my work so no one could do anything. So, that is, of course, the downside of the thing... [Participant 4, 59-year-old woman, focus group 4]
- I guess now—real nice if sometimes one could be reached out to from there by email or something else... [Participant 9, 44-year-old man, focus group 2]
- I’m waiting for it and I’m truly interested, as if I were waiting for something like a spark. That it is something, something like, motivating... [Participant 56, 45-year-old man, focus group 3]

## Results

**Figure 1:**
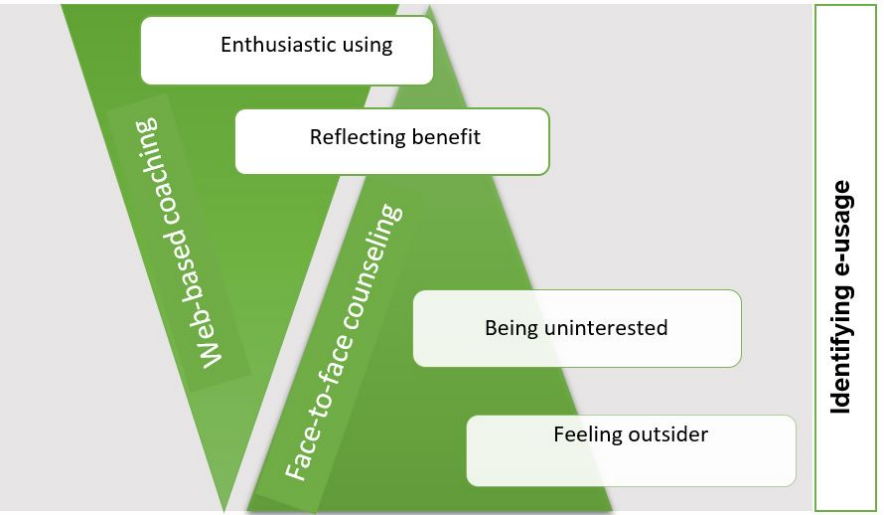
Cardiac rehabilitees’ different experiences of using technology and expectations of remote counseling.

The descriptors of the rehabilitees’ prior technology experience are “feeling outsider,” “being uninterested,” “reflecting benefit,” and “enthusiastic using.” The category “identifying e-usage” describes the essence of the rehabilitees’ experiences with using technology and identifying its usage (Figure 1). Individuals in the “feeling outsider” and “being uninterested” subcategories need more face-to-face counseling, while Web-based coaching is sufficient for the individuals in the “reflecting benefit” and “enthusiastic using” categories. These conclusions are based on the following results.

The first subcategory, “feeling outsider,” consists of rehabilitees who fear that they do not have sufficient skills as computer users to participate in remote rehabilitation. They have not used the computer at all or they have only basic computer skills. In the following samples, two rehabilitees discuss “feeling outsiders.”

Because I don’t have a computer, I am a total outsider. So...because of this, I’m not so terribly interested. The only thing I know about this is that the self-tracking is great.

Participant 39, 74-year-old man, focus group 1

That technology hasn’t really come...My wife taught the computer...supported, well, taught—so I went to the courses. And the kids did. I thought that if I’m still starting to tinker, there won’t be enough hours in the day to learn.

Participant 25, 60-year-old man, focus group 1

These rehabilitees feel they do not have adequate computer skills, and few of them use computers at work. If they need help with technology, they request it from friends or family members. They feel they have no time to study computer use, and the English language is also difficult. Concerns about the impact of technology on health and security seem to be other reasons for avoiding computer use. In the following sample, a rehabilitee discusses his learning experiences with information technology.

Better that it leaves you, like if you go the bank computer, then everything gone. It doesn’t help there. Still it doesn’t. This isn’t the only reason, but...I'm not interested. I’ve taken two computer courses, though. Last time I went to apply for a bus-driving license, and it was two weeks. And when I went and when I came back I was as dumb as I going there, and I come back...Of course, I know the time to buy one is coming, but I’m holding it off for as long as I can.

Participant 58, 63-year-old man, focus group 4

The following rehabilitee has not yet established expectations for counseling.

I’m not really sure...waiting to see what comes.

Participant 64, 64-year-old, man, focus group 2

**Figure 2:**
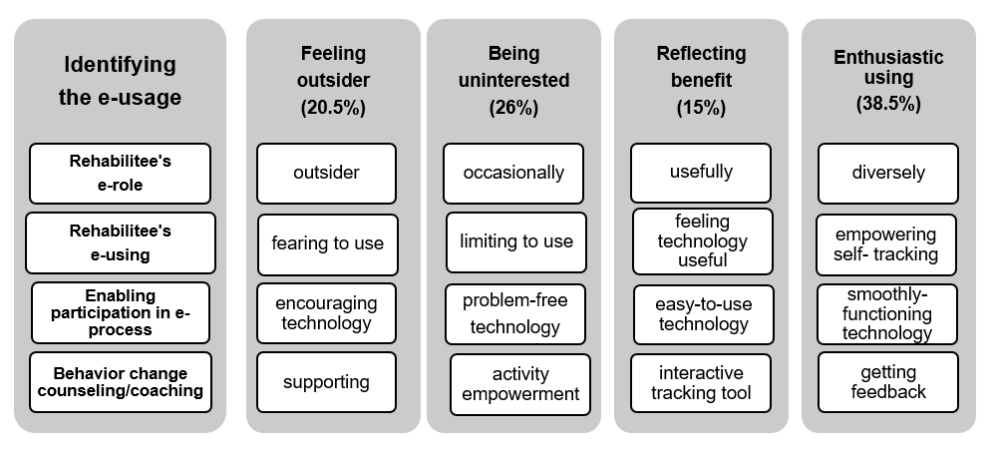
Identifying the e-usage category and subcategories of technology users.

For these rehabilitees, technology is something terrifying and almost incomprehensible. They are aware of its applications, such as Facebook, but these applications are foreign to them and therefore they feel like outsiders. Nevertheless, their positive expectations toward technology encounters are apparent although they do not expect anything amazing from Web-based counseling. They need guidance to support them in the use of technology (Figure 2). Figure 2 is a summary of the “identifying e-usage” category, which was created based on the rehabilitees’ e-role and e-usage. The subjects in the “feeling outsider” subcategory regard themselves literally as outsiders and find technology fearsome; on the other hand, they look forward to overcoming this fear and expect adequate support.

The second subcategory is “being uninterested.” It is based on the experiences of rehabilitees who are conversant with technology and have experimented with social media. Their experiences are limited to necessary and occasional uses such as paying bills, renewing book loans, and reading emails. If they encounter a technical problem, their interest fades. They are not interested in using technology to connect socially via email and social media. They are worried about information security. In the following sample, a rehabilitee explains why he is not interested in technology.

I’m not terribly interested in that remote stuff, because...well, I use the computer at work every day...That email reading, I may go and check my mail once a week. It’s not in a way...maybe it just isn’t my thing...it’s no trouble to surf on the internet in the evening. Only when I must. I can check email—if a bill has arrived, I can pay it there.

Participant 9, 44-year-old man, focus group 2

The following rehabilitee discusses problems related to technology and social media.

...I’ve noticed sometimes that when I’ve been at it for some time, the machine has broken down in the middle of my work so no one could do anything. So, that is, of course, the downside of the thing...

Participant 5, 53-year-old woman, focus group 4

But then what really irritates and frustrates me and just can’t interest me—although I’m there in Facebook because my nephew forced me there.

Participant 5, 53-year-old woman, focus group 4

The following rehabilitees discuss coaching.

I’m waiting for it and I’m truly interested, as if I were waiting for something like a spark. That it is something, something like, motivating, and...well...I can’t say, but it like maybe not now for sure every week. If once a month, certainly something could come...a reminder.

Participant 56, 45-year-old man, focus group 3

When I could enter inputs in there, and if my own activities could be there, then I would be like a response: Is this the right or wrong direction, and...And that’s when it’s really somebody, something and someone monitoring what you’re doing.

Participant 41, 49-year-old woman, focus group 2

These rehabilitees use technology occasionally and their daily use is limited. They value problem-free technology. They expect activity empowerment counseling, which should motivate and spark interest, but believe that technology demands a coach who would give feedback, assign weekly tasks, and issue regular reminders. This subcategory includes “occasionally,” “limiting to use,” “encouraging technology,” and “supporting counseling” (Figure 2).

The subjects in the third subcategory, “reflecting benefit,” maintain an interest in technology for only as long as they have an indispensable need for it in everyday life. In the following, a rehabilitee discusses his use of technology in free time.

Just like the pharmacy, like in that do I medications? In that case, is the prescription still valid? And like that, just in that way...Yeah, it is good to look...there are plenty of medicines left, and...Do I have to bring in, or order a new prescription? And other things, just in that way. What now happens every day or when it’s needed so...so I don’t go surfing on every webpage...Yes, with the children I use it, and with friends I like to connect over the internet.

Participant 24, 65-year-old man, focus group 4

The experiences of these persons fall into two groups. The subjects in the first group find technology difficult and need time to learn it. For example, they may find remembering passwords difficult. They need help to learn security procedures and computer usage.

And for paying bills I use it most, too. Some information when it’s needed, well yes, I try to find it from there then. And if someone wants to find the frustrating side, well, those passwords frustrate me, because they always go missing...and a password has gotten lost, and...I can’t go there anymore. Of course I can create a new password, but it is such a bother—just forget about it. And I read magazines in the computer, and...

Participant 36, 68-year-old man, focus group 4

Even though the use of technology is not a problem to the subjects in the second group, they still eschew technology. Since they see technology as something negative, they use it only when necessary—for example, to search for information. Some rehabilitees had experience in digital physical activity monitoring. In the following example, a rehabilitee discusses the usability of technology.

...But then the computer, when it runs all day—I don’t want that. That’s why I don’t open the computer in the evening...Of course it’s easy always that everything could be, like as easy-to-use as possible, because that’s why I don’t do it, when I could use it for remote technology. But it should be as easy-to-use as possible: it should be as automatic as possible, this thing. It, I think should be as flexible as possible.

Participant 17, 57-year-old man, focus group 2

In the next example, a rehabilitee discusses self-monitoring and coaching.

Let’s put it in this way: I’m not actually now that way from being pushed, yeah. Yes it comes from my own desire. The main purpose is monitoring: it’s for that. It’s interesting to follow what happens if you change some exercise habits, and you can see from this, what changes have happened in the background. Very okay.

Participant 17, 57-year-old man, focus group 2

This subcategory, “reflecting benefit,” emerged with four properties: “useful,” “feeling technology useful,” “easy-of-use technology,” and “interactive tracking tool” (Figure 2). These rehabilitees expect technology to be easy to use and also expect it to enable communication. Essentially, they do not need a coach but they need tools for self-monitoring and helping to improve their health.

The subjects in the fourth subcategory, “enthusiastic using,” show a positive attitude toward technology and have used it for a long time in a variety of ways, both in everyday life and at work through mobile phones, tablet computers, and desktop computers. In the following examples, enthusiastic users discuss the use of technology.

Well, laptops are always open less when you have a smartphone. In other words, I read those emails easily on my phone. Therefore I don’t turn on the laptop.

Participant 8, 61-year-old woman, focus group 2

More there is, of course, invoice writing and information retrieval, but of course electrical diagrams, and...Sometimes some programming, logic, some small logic programming, and something like that.

Participant 20, 64-year-old man, focus group 3

These persons follow emails actively through mobile phones. Many of them have mobile health and exercise activity apps such as the Sports Tracker (Sports Tracking Technologies). They use social media such as Facebook and WhatsApp to keep in touch with friends and relatives. They do not consider internet problems particularly annoying and contact a specialist if they find this necessary. They are interested in technology and want to develop their technology skills. In the following example, rehabilitees describe what they expect from technology and from a coach.

I’m waiting and I’m interested. Yes, of course, this here now gives a little push in the pants. I’m already moving pretty well, that’s what this thing around my arm tells me...Yeah...and then yes, I have the Sport Tracker on my phone, also. When I go somewhere, I tell it to draw a map, and I see the time and all that.

Participant 66, 34-year-old man, focus group 3

Modern opportunities. And if now, of course...from where soon could come a little spark, and that spark continues than exercise could begin. And it’s really the same benefit. And then, of course, if nothing’s heard from there. It sounds real good, and then reminders. Something like you can write comments, and...

Participant 26, 61-year-old woman, focus group 2

Maybe this is kind of a simple-enough device. When there’s not anything amazing in here now, then owing to that, it’s comfortable to use: It’s not too complicated.

Participant 15, 52-year-old man, focus group 1

These rehabilitees use technology diversely and effortlessly, also for self-tracking. They expect Web-based intervention to be simple, motivating, easy to use, and interactive. They also expect coaches to give feedback if anything is missing. The attitude among the subjects in this subcategory is best described with the phrases “diversely” and “empowering self-tracking,” and the best descriptors of expectations toward technology and counseling are, respectively, “smoothly functioning” and “getting feedback” (Figure 2).

## Discussion

### Principal Findings

The study shows that the diversity of the rehabilitees as technology users and their different needs for technology should be taken into account in rehabilitation planning. The four subcategories are “feeling outsider,” “being uninterested,” “reflecting benefit,” and “enthusiastic using” (Figure 2). Some rehabilitees whose e-role is “outsider” or “occasionally using” need face-to-face communication for a large part of rehabilitation, while individuals whose e-role is “usefully” or “diversely” need incrementally less face-to-face interaction and feel that Web-based behavior change coaching will provide sufficient support for their rehabilitation. However, all rehabilitees hope that technology would be simple, flexible, and easy to use and learn, which would enable participation in an e-process. Participation in a remote rehabilitation program, as in this research, requires skills in areas such as Web-based log-ins and in reading and responding to tasks and messages. Activity self-monitoring requires downloading an activity tracker program to the computer and synchronizing the program with the computer.

The rehabilitees in the “feeling outsider” subcategory have a positive attitude toward technology, yet they do not see technology important for themselves. Their mindset supports previous research results that positive attitudes toward technology is a prerequisite for the uptake of technology [29,30] and eHealth [19]. At the beginning of remote rehabilitation, it is important to encourage rehabilitees’ abilities to use various devices, since these abilities will make them more receptive to the use of technology [31] and enable participation in remote rehabilitation. Studies have also found that even though the digital skills of senior citizens have improved, they are still insufficient [32]. Rehabilitees’ ability to use technology is also ensured by adequate internet technology support in remote rehabilitation [33]. Finally, it is important that apps and instructions are available in the user’s own language.

The rehabilitees in the “being uninterested” subcategory were not interested in technology and eschewed its use. They felt that easy-to-use technology encourages technology use while technology that does not work frustrates, and they felt constant communication in social media irritating. They expect coach contact to maintain motivation during remote rehabilitation. However, when their expectations of technology are exceeded, the resulting experience is positive and pleasant [34,35] and maintains motivation [35], which has also been shown in previous research.

The rehabilitees in the “feeling outsider” and “being uninterested” subcategories need more face-to-face counseling during remote rehabilitation. The rehabilitees in the “feeling outsider” subcategory need supportive guidance in technology use, while the individuals in the “being uninterested” subcategory need to be motivated in order to create positive experiences. Maintaining a spark of interest and motivation requires a motivator and a coach who gives feedback, weekly assignments, and regular reminders [36]. Previous research has shown that interventions based on the behavior change theory may motivate more than those lacking a theoretical basis, but studies conducted on mobile cardiac rehabilitation have not specifically addressed behavior change strategies. Web-based interventions may provide an opportunity for real-time coaching [37,38], motivation, and engagement, allowing rehabilitees to achieve a meaningful behavior change [18]. The rehabilitees feel that they need an external motivator, and the importance of the behavior change theory should therefore be given an adequate emphasis in the planning of remote rehabilitation.

The rehabilitees in the “reflecting benefit” subcategory use technology daily, and technology challenges they encounter stem from technical problems and attitudes. They expect apps to be easy to use, secure, and in their own language. The perceived ease of technology use influences perceived usefulness and together these bolster their intention to use technology for a real purpose (usage behavior) [39]. The technology acceptance model has also been applied in the health care context [34,35]. In addition, perceived usefulness with a perceived value plays a role in the acceptance of technology [40]; for example, it provides personalized information, support, monitoring, and feedback [21,22]. The minimization of application risks increases trust in systems [21,33,35]. These rehabilitees’ acceptance of technology increases when applications are easy to use and interactive, which has also been shown in previous qualitative studies.

Remote rehabilitation should enable social participation, such as peer group discussion and personalized feedback. These rehabilitees use social media as a means of communication and appreciate the possibility to interact. Social media, such as Facebook, Twitter, Pinterest, and Instagram, is part of their day-to-day life [41]. Social participation should be used in remote rehabilitation by granting them access to a peer group [16,17] and enabling problem-free peer group discussion on matters regarding the rehabilitation process. Health care rehabilitation applications allow users to receive information and interact since rehabilitees can receive assignments, record and review data, receive automated feedback, and connect with peers or health care professionals. All of these have been found to be important Web-based user experiences in previous studies [20-22,38]. These rehabilitees’ experiences show little need for other services than automated feedback in the form of mainly interactive coaching, which gives a little push and supports a lifestyle change.

The rehabilitees in the “enthusiastic using” subcategory accept technology as an integral part of their everyday life. Statistics show that mobile phone use is increasing [42], and these rehabilitees use mobile phones actively. Mobile health apps are increasingly popular, and mobile phone users have downloaded mobile health apps [43]. They are keen users of sufficiently coach-supported Web-based intervention apps to boost motivation for physical activity [14,44,45]. Recent studies show that participants appreciate professional Web-based support [20]. The subjects of these studies had participated in Web-based e-rehabilitation, which reduces face-to-face interactions [45,46] and is particularly suited to an active user who adequately masters technology and is interest in it. Easy-to-use and smoothly functioning technology allows extensive personal activity and body function monitoring—in other words, self-tracking. The recently termed quantified self notion has emerged to promote self-knowledge through numbers [9]. Health change coaching is based on the behavior change theory, motivational strategies, and communication techniques [20,46].

The rehabilitees’ experiences with and attitudes toward technology provide information on how to implement a counseling theory and methods for the planning of remote rehabilitation. A coach should conduct individual risk factor assessment and management, exercise training, and self-management of modifiable risk factors and provide education and psychosocial support [38]. Professional health coaches can help rehabilitees increase self-direction, set specific goals, and take action to achieve and sustain health-supporting behaviors [38,47-49]. In addition to self-monitoring, an easy-to-use interface is a desirable feature in mobile apps for promoting physical activity. Examples of these interfaces are the integration of biosensors that collect information from body and life systems such as electrocardiogram, physical activity, heart rate, blood pressure, and blood glucose measurement [50]. Digital stethoscopes, thermometers, and weight scales [9] can also be used in remote rehabilitation.

The topic is important because remote rehabilitation is already being implemented and will continue to be implemented in the future due to increasing digitization [46,47]. Technology-related studies have shown that remote technology is most successful when it is simple and designed for easy understanding and easy use [30]. Easy-to-use technology also produces positive and successful experiences [49]. As rehabilitees’ skills develop, they gain self-esteem and are empowered to expect positive, successful experiences [29,32]. Remote rehabilitation must take into account the abilities of each rehabilitee in learning, cognition, and motor and perceptual skills [29,33,51] and allow an individual ample time to master new skills [29]. In the future, the recognized needs and concerns of the subjects in the “feeling outsider,” “being uninterested,” “reflecting benefit,” and “enthusiastic using” categories should be combined with previous research and taken into account. In this way, the acceptance and use of remote technology could be upped for more meaningful and effective rehabilitation. The results of this study can also be used in designing remote rehabilitation and health coach training programs. There is a lack of quality research on the experiences of coronary disease patients, and a need exists for mixed-methods research for the development of easy-to-use effective and meaningful welfare technology.

### Limitations

There are weakness that need to be considered when interpreting the findings of this study. The subjects discussed their experiences of technology at the beginning of rehabilitation, and everyone was given the opportunity to share his or her experiences. The interviewer created an accepting atmosphere and encouraged silent participants. Despite this, it is possible that the participants were trying to please the group when answering the questions. On the other hands, there are advantages. The results have attracted interest and their relevance, credibility, and usefulness have been identified as important when implementing remote rehabilitation. They have been also used in comparative rehabilitation groups, in musculoskeletal system reconditioning, and in work ability rehabilitation.

### Conclusions

The aim of this study was to explore in detail rehabilitees’ experiences and attitudes toward technology. The results are the rehabilitees’ technology experiences described as “feeling outsider,” “being uninterested,” “reflecting benefit,” and “enthusiastic using,” which relate to their e-usage. The results help providers and health workers to identify different technology users among potential rehabilitees and determine what use levels must be taken into account when developing remote rehabilitation. The category formed into four subcategories which define the rehabilitees’ technology experience and attitude. The results can also be used more widely in different contexts of social and health care for the planning of and training in remote rehabilitation/e-rehabilitation counseling and education.
